# Tigecycline Treatment for Carbapenem-Resistant *Enterobacteriaceae* Infections: A Systematic Review and Meta-Analysis

**DOI:** 10.1097/MD.0000000000003126

**Published:** 2016-03-18

**Authors:** Wentao Ni, Yuliang Han, Jie Liu, Chuanqi Wei, Jin Zhao, Junchang Cui, Rui Wang, Youning Liu

**Affiliations:** From the Department of Respiratory Diseases (WN, CW, JZ, JC, YL), Chinese PLA General Hospital; Department of Neurology (YH), Chinese PLA 305 Hospital; Department of Vascular and Endovascular Surgery (JL); and Department of Clinical Pharmacology (RW), Chinese PLA General Hospital, Beijing, China.

## Abstract

Carbapenem-resistant *Enterobacteriaceae* (CRE) infections are prevalent worldwide; they have few effective treatments and this jeopardizes public health. Clinicians often use tigecycline to combat CRE, but its clinical efficacy remains controversial. Therefore, to compare the efficacy and safety of tigecycline in treating CRE infections compared with that of other antimicrobial agents, and to evaluate whether combination therapy and high-dose regimens are beneficial, we performed a systematic review and meta-analysis.

PubMed and Embase were searched for controlled trials or cohort studies reporting the efficacy and/or safety of tigecycline-based regimens to treat CRE infections. Statistical analyses were performed using the Comprehensive Meta-Analysis V2.2. All meta-analyses were performed based on fixed- or random-effects model, and the *I*^*2*^ method was used to assess heterogeneity.

Twenty-one controlled studies and 5 single-arm studies were included in this systematic review. With regard to the controlled studies, the tigecycline groups did not differ significantly from the control groups in terms of overall mortality (Odds ratio (OR) = 0.96 [95% confidence interval (CI) = 0.75–1.22; *P* = 0.73]), clinical response rate (OR = 0.58 [95% CI = 0.31–1.09; *P* = 0.09]), or microbiological response rate (OR = 0.46 [95% CI = 0.15–1.44; *P* = 0.18]). Subgroup analyses showed that 30-day mortality was significantly lower in patients who received tigecycline combination therapy than in those who received monotherapy (OR = 1.83 [95% CI = 1.07–3.12; *P* = 0.03]) and other antibiotic regimens (OR = 0.59 [95% CI = 0.39–0.88; *P* = 0.01]), respectively. In addition, high-dose tigecycline regimens differed significantly from standard dose schedules in terms of ICU mortality (OR = 12.48 [95% CI = 2.06–75.43; *P* = 0.006]). The results of the 5 single-arm studies corroborated the findings of the controlled studies.

Our results indicated that the efficacy of tigecycline in treating CRE infections is similar to that of other antibiotics. Tigecycline combination therapy and high-dose regimens may be more effective than monotherapy and standard-dose regimens, respectively. Nonetheless, considering that the current available evidence is limited, well-designed randomized controlled trials are urgently needed to clarify the comparative efficacy of tigecycline in treating CRE infections.

## INTRODUCTION

*Enterobacteriaceae*, such as *Klebsiella pneumoniae, Escherichia coli*, and *Enterobacter cloacae*, are frequently involved in hospital-associated infections. In particular, strains that produce extended-spectrum β-lactamases are common.^1^ Carbapenems are the most broadly used first-line antibiotics for such infections. However, widespread use of these drugs has resulted in the emergence of carbapenem-resistant strains, most of which produce carbapenemases and are, therefore, resistant to the drug.^2^ In recent years, these versatile carbapenemases have spread worldwide among the *Enterobacteriaceae*, especially *K pneumoniae*. For this reason, nosocomial outbreaks of carbapenem-resistant *Enterobacteriaceae* (CRE) are frequent worldwide, leading to prolonged hospital stays and higher mortality rates.^3^

As these multiple resistant strains can acquire resistance to nearly all classes of antibiotics available in the clinic, selection of the appropriate antimicrobial treatment has become difficult. In fact, such limitations have forced clinicians to reuse polymyxins, a group of polypeptide antibiotics discovered in the 1940s.^4^ However, the severe nephrotoxicity of these drugs contraindicates their use in many cases, especially among critically ill patients with renal insufficiency.^5^ Tigecycline, the first member of the glycylcycline class of antibiotics, has shown promising in vitro activity against CRE.^6^ It binds with high affinity to bacterial ribosomes and is unaffected by the typical mechanisms that render bacteria resistant to the tetracycline class.^7^ Several clinical studies have investigated the efficacy of tigecycline in treating CRE infections; yet these have yielded variable results. Suboptimal concentrations of the drug have been found in both serum and pulmonary epithelial lining fluid, and this has prompted many physicians to use either combination therapy or high-dose tigecycline to treat CRE infections.^8–10^ However, whether combination therapy or high-dose regimens are more effective is not clear.

Therefore, we performed a systematic review to compare the efficacy and safety of tigecycline with those of other antimicrobial agents in treating CRE infections, as well as to evaluate whether combination therapy and high-dose regimens are beneficial.

## METHODS

### Literature Search

We searched PubMed and Embase from their inception until September 20, 2015. The main search terms were: “escherichia,” “klebsiella,” “enterobacter,” “proteus,” “serratia,” “citrobacter,” “salmonella,” “shigella,” “enterobacteriaceae,” and “tigecycline.” Furthermore, the reference lists of all identified reports were hand-searched for relevant articles. No language restrictions were applied.

### Study Selection Process

The Preferred Reporting Items for Systematic reviews and Meta-analysis statement were strictly followed. Papers were considered eligible if they were controlled trials or cohort studies reporting the efficacy and/or safety of tigecycline-based regimens to treat carbapenemase-producing *Enterobacteriaceae* and/or CRE. Investigations that focused on laboratory research or epidemiology, and case reports or series that included < 10 infected patients treated with tigecycline were excluded. The literature search and study evaluation were separately performed by 2 investigators (Ni and Han), and any disagreements were resolved by the third author (Liu).

### Ethical Review

Ethical approval was not required in this study.

### Data Extraction and Quality Assessment

Two reviewers independently extracted data and assessed the risk of bias. The following data were extracted from each study: (1) authors and year of publication; (2) study design; (3) baseline characteristics of the study population (sample size, age, sex, underlying conditions, and severity of illness based on ICU admission and APACHE score); (4) coadministration of other antibiotics; (5) type of microorganism; (6) outcomes, including mortality, such as the mortality of 14-day, 30-day, in-hospital, ICU, and CRE infection-related (death mainly attributed to the CRE infections), clinical response, and microbiological response; (7) reported adverse effects; and (8) emergence of resistance during treatment.

We used the modified Newcastle–Ottawa scale (NOS) to assess the quality of the included studies.^11^ Studies with an NOS score <3 were considered poor quality and excluded from this meta-analysis.

### Definitions and Statistical Analysis

Because of the high mortality rates among patients with CRE infections, we chose mortality as the primary outcome. The secondary outcomes were: clinical response, microbiological response, adverse effects, and emergence of resistance. Microbiological response was defined as successful when eradication or sterile culture results were obtained during or after the antibiotic therapy. Because there are no standard criteria to assess clinical response and adverse events, we accepted the criteria as reported in each study.

All statistical analyses were performed using the Comprehensive Meta-Analysis V2.2 (BioStat, Englewood, NJ). In studies that provided only median and range for continuous outcomes, mean value and variance were estimated using the median and the range.^12^ Among the controlled studies, the between-study heterogeneity was assessed using the *I*^*2*^ test, whereby *I*^*2*^ values >50% were defined as indicating heterogeneity. Either fixed-effects (Mantel–Haenszel method) or random-effects (DerSimonian and Laird method) models were used, depending on the heterogeneity result. Binary outcomes from controlled studies were expressed as odds ratios (ORs) with their 95% confidence intervals (CIs), and continuous outcomes were expressed as the mean difference between 2 groups. Egger regression, as well as the Begg and Mazumdar methods, was used to evaluate publication bias. In single-arm studies, the between-study heterogeneity was assessed using the *Q*-statistic method, and random-effects models were used to pool data. *P* values < 0.05 were considered statistically significant.

## RESULTS

### Included Studies

**FIGURE 1:**
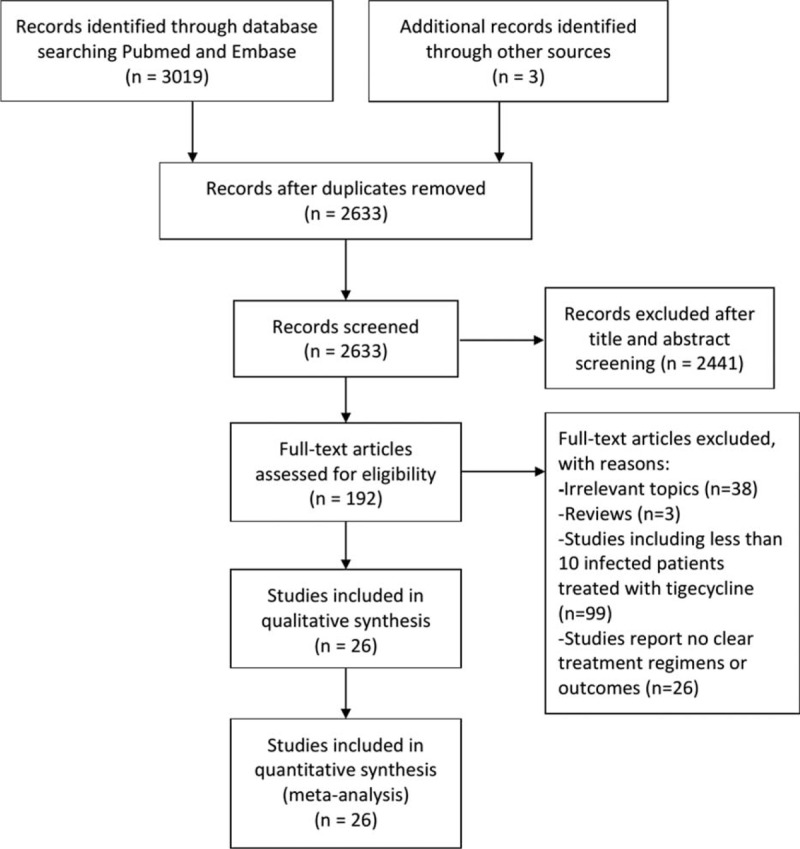
Flowchart of the article selection process.

The literature search identified 3019 citations from the 2 databases, plus 3 studies identified from references. In total, 192 full-text articles were screened. Ultimately, 21 controlled studies (1595 patients)^13–33^ and 5 single-arm studies (113 patients)^34–38^ were included in this systematic review. The search and study selection process is detailed in Figure 1.

### Study Characteristics

Table 1  shows the characteristics of the studies included in this systematic review shows. All 21 controlled studies were not randomized controlled trials (RCTs). Six of them were prospective cohort studies, 14 were retrospective studies, and 1 was an ambispective study. All of the included controlled studies had an NOS score > 3. Two of the single-arm studies were prospective studies, and the others were retrospective studies. Most patients in the included studies were critically ill, with 51.6% of them in ICU. Seventeen studies reported the patients’ APACHE scores, with an average value of 19.0. Eleven studies addressed CRE infections, and 15 pertained to carbapenemase-producing *Enterobacteriaceae* infections. *Klebsiella* spp were the major causative pathogen, and bacteremia was the most common manifestation; this was followed by urinary tract infection and nosocomial pneumonia.

**TABLE 1:**
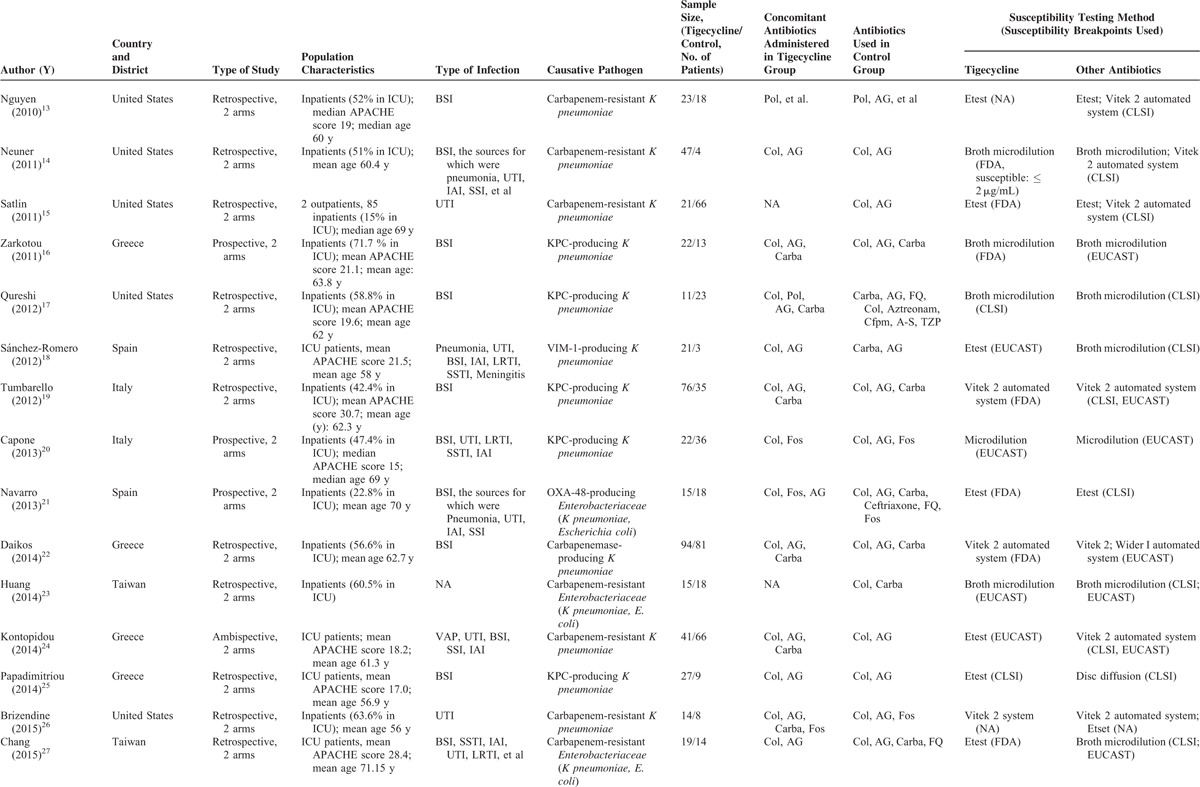
Characteristics of Studies Included In Systematic Review and Meta-analysis

**TABLE 1 (Continued):**
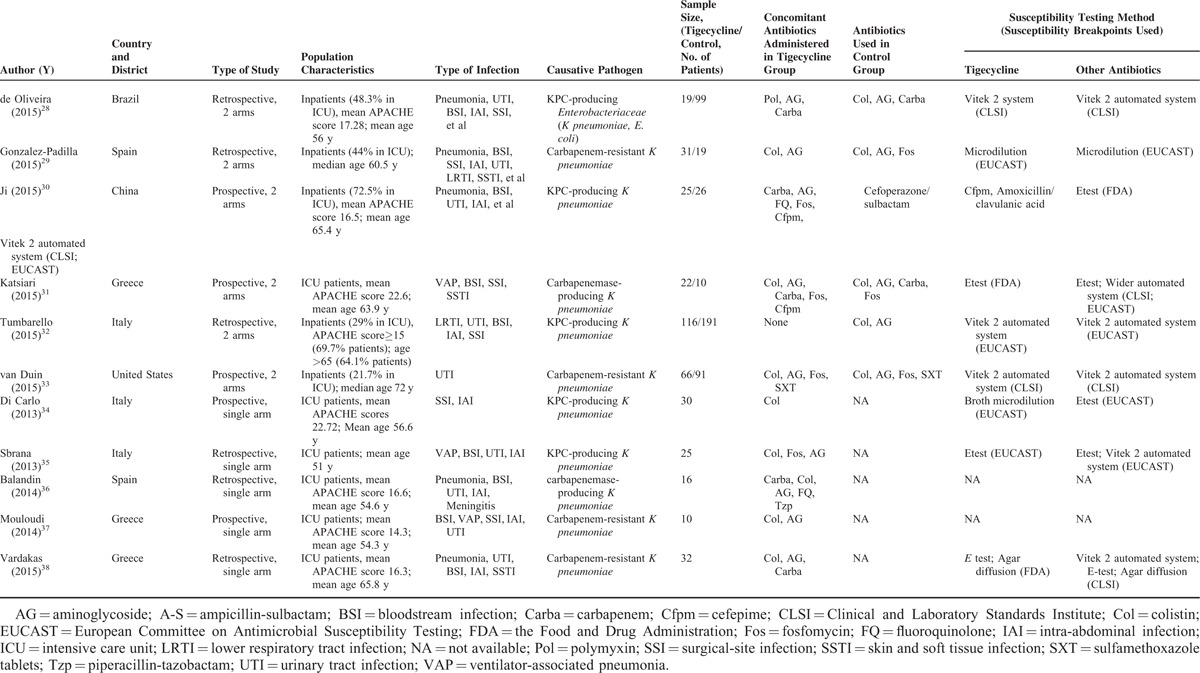
Characteristics of Studies Included In Systematic Review and Meta-analysis

### Mortality

**FIGURE 2:**
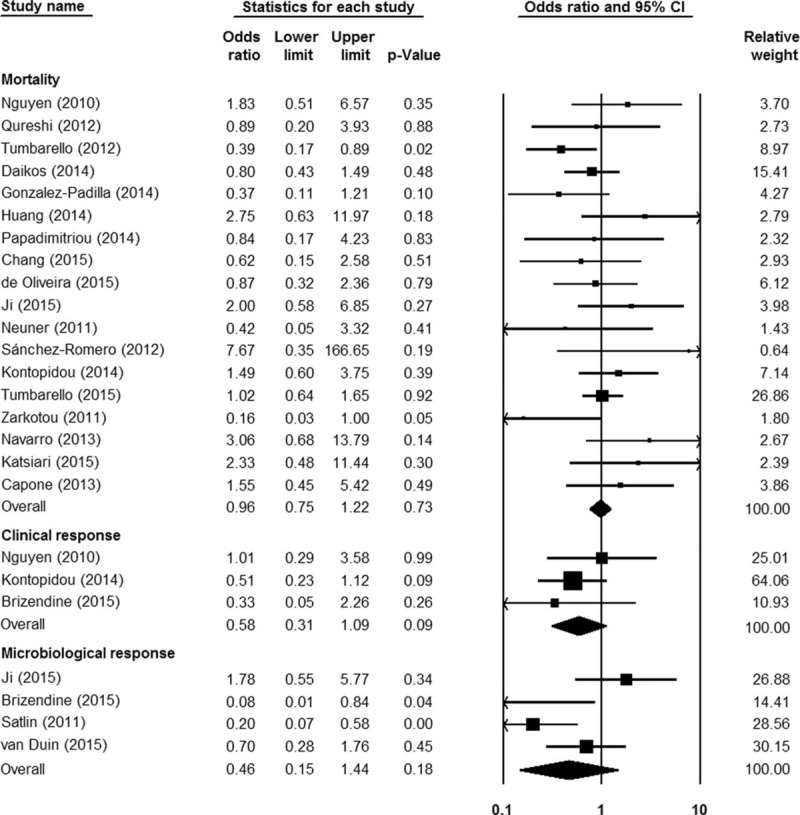
The efficacy of tigecycline, as compared with other antibiotics, in treating infections caused by carbapenemase-producing *Enterobacteriaceae* and carbapenem-resistant *Enterobacteriaceae*.

**FIGURE 3:**
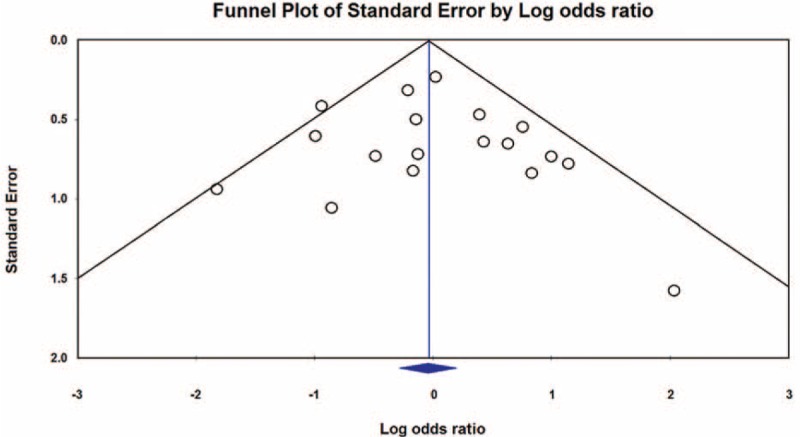
A funnel plot of mortality rate in patients treated with tigecycline, as compared with that in patients treated using other antibiotics, for infections caused by carbapenemase-producing *Enterobacteriaceae* and carbapenem-resistant *Enterobacteriaceae*.

As shown in Figure 2, tigecycline groups did not differ significantly from control groups in terms of overall mortality (18 studies; 1328 patients; OR = 0.96 [95% CI = 0.75–1.22; *P* = 0.73]). Because of the low statistical heterogeneity among the studies (*I*^*2*^ *=* 26.95%; *Q* = 23.27 [*P* = 0.14]), a fixed-effects analysis model was used. No significant statistical bias was detected by either Egger regression (*t* = 0.68; *df* = 16.0; *P* = 0.51) or Begg and Mazumdars rank correlation (Kendall *τ* = 0.14; *P* = 0.40). The funnel plot for publication bias demonstrated no evidence of asymmetry, as shown in Figure 3. Tables 2 and 3 show the subgroup analysis of the controlled studies. The tigecycline monotherapy group did not differ significantly from the controls in terms of mortality; however, a significant difference with respect to 30-day mortality was observed between the tigecycline combination therapy group and the controls.

**TABLE 2:**
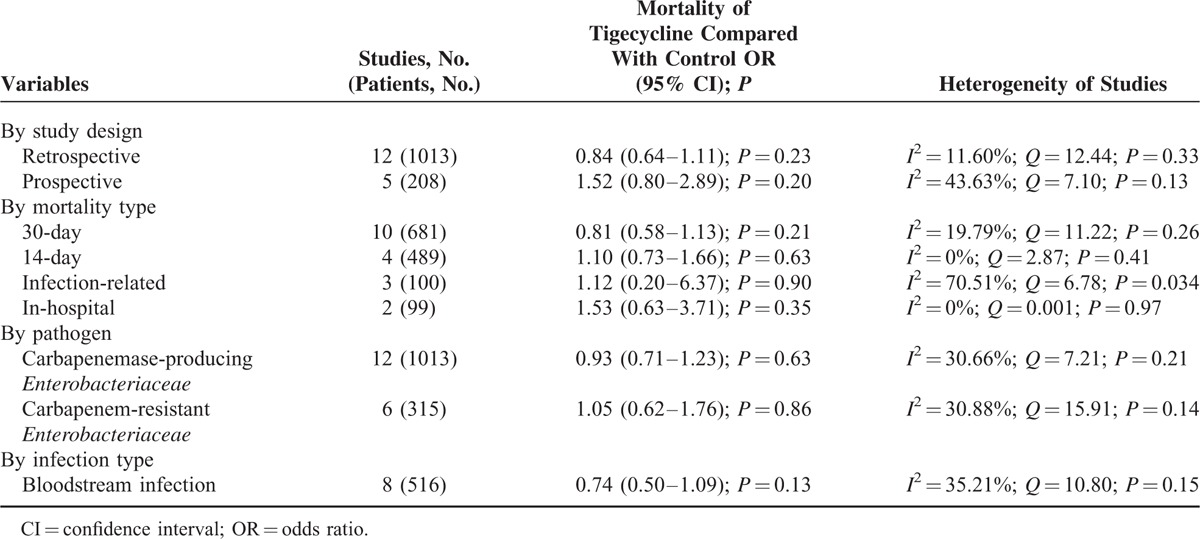
Subgroup Analysis of Overall Mortality With Tigecycline Versus Other Antibiotics for Treatment of Carbapenem-Producing *Enterobacteriaceae* and CRE Infections in Controlled Studies

**TABLE 3:**
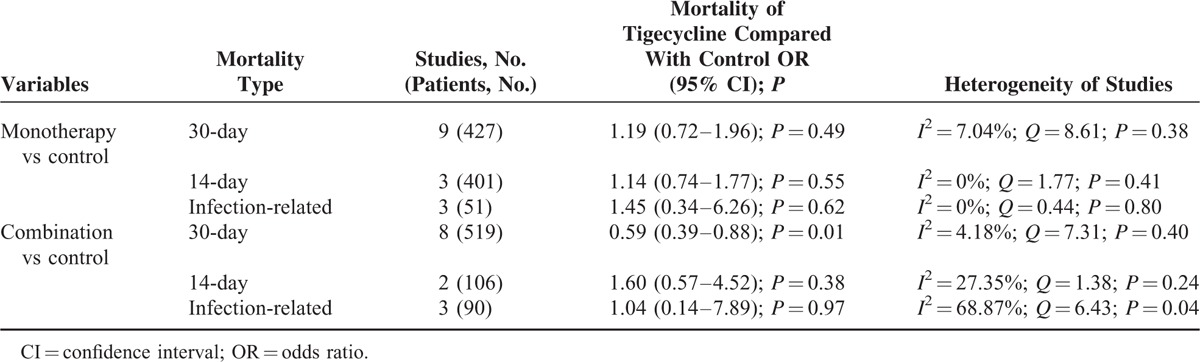
Subgroup Analysis of Mortality With Tigecycline Monotherapy or Combination Therapy Versus the Controls for Treatment of Carbapenem-Producing *Enterobacteriaceae* and CRE Infections in Controlled Studies

In the 5 single-arm studies, the pooled overall mortality rate was 39.21% (95% CI = 0.19–0.65; *I*^2^ = 81.16%; *Q* = 21.23 [*P* < 0.001]), which corroborated the results of the controlled studies (36.3% [95% CI = 0.32–0.41; *I*^2^ = 31.58%; 43%; *Q* = 24.85 [*P* = 0.10]).

Table 4 displays the subgroup analysis of the different tigecycline treatment regimens. The 30-day mortality in the combination therapy group was significantly lower than that in the monotherapy group. The tigecycline triple combination differed significantly from the double combination, and the high-dose regimen was significantly different from the standard-dose regimen. In addition, a significantly higher 30-day mortality was noted in the monotherapy group than in the combination therapy group in cases of blood stream infection (OR = 2.12 [95% CI = 1.17–3.86; *P* = 0.01]; *I*^2^ = 35.73%; *Q* = 10.89 [*P* = 0.14]).

**TABLE 4:**
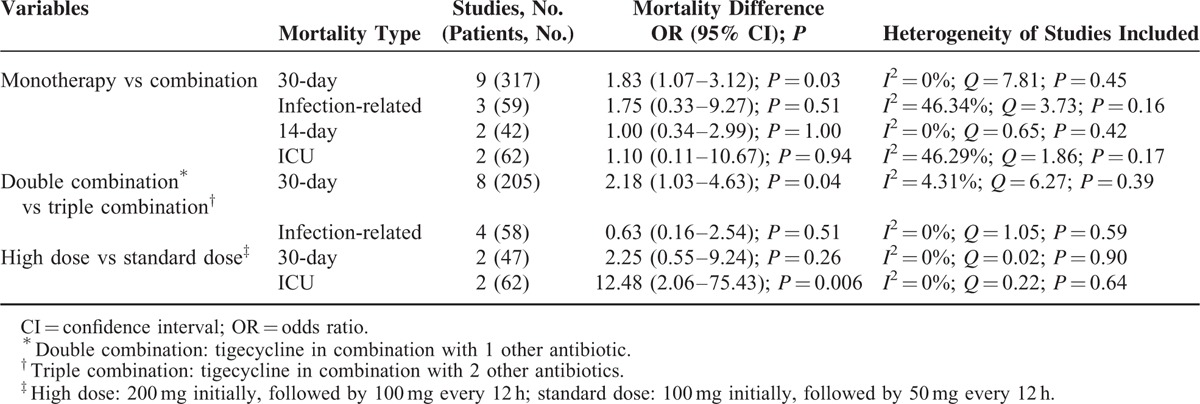
Subgroup Analysis of Mortality Using Different Tigecycline Regimens to Treat Carbapenem-Producing *Enterobacteriaceae* and Carbapenem-Resistant *Enterobacteriaceae* Infections

### Clinical Response

Three controlled (170 patients) and 2 single-arm (42 patients) studies addressed the clinical response after treatment. No significant differences were observed between the tigecycline and control groups in this regard (OR = 0.58 [95% CI = 0.31–1.09; *P* = 0.09]; *I*^2^ = 0%; *Q* = 1.167 [*P* = 0.56]; Figure 2). In the single-arm studies, the pooled clinical response was 49.68% (95% CI = 0.19–0.81; *I*^2^ = 72.46%; *Q* = 3.63 [*P* = 0.06]). In subgroup analysis, tigecycline monotherapy did not differ significantly from combination therapy in this regard (OR = 0.55 [95% CI = 0.18–1.72; *P* = 0.30]; *I*^2^ = 0%; *Q* = 0.69 [*P* = 0.71]).

### Microbiological Response

Four controlled studies (316 patients) demonstrated the comparison of tigecycline groups with control groups in terms of microbiological response, and no significant differences were observed between the groups (OR = 0.46 [95% CI = 0.15–1.44; *P* = 0.18]; *I*^2^ = 70.53%; *Q* = 10.18 [*P* = 0.017]; Figure 2). The pooled microbiological response rate of the single-arm studies (2 studies, 24 patients) was 51.81% (95% CI = 0.21–0.81; *I*^2^ = 61.77%; *Q* = 2.62 [*P* = 0.11]). Six studies (149 patients) reported comparison of comparison of tigecycline monotherapy with combination therapy in terms of microbiological response; no significant differences were found between the 2 groups in this regard (OR = 0.48 [95% CI = 0.19–1.20; *P* = 0.12]; *I*^2^ = 0%; *Q* = 4.08 [*P* = 0.54]).

### Adverse Effects and Emergence of Resistance

Three single-arm studies monitored adverse effects. The common adverse effects of tigecycline (nausea, vomiting, and diarrhea) were not mentioned in any of the studies. In 1 study (30 patients), the minimum inhibitory concentrations (MICs) of tigecycline in 5 different KPC-producing *K pneumoniae* strains increased from ≤ 0.5 μg/mL to 0.8 to 1.0 μg/mL after tigecycline treatment.

## DISCUSSION

Tigecycline has been approved by the United States Food and Drug Administration to treat complicated skin and skin structure infections, complicated intra-abdominal infections, and community-acquired pneumonia. In contrast, the present systematic review indicated that bacteremia was the most common manifestation of CRE infection, followed by urinary tract infection and nosocomial pneumonia. Thus, the use of tigecycline to treat CRE infections can be described as off-label. A previously published meta-analysis indicated that tigecycline is no more effective than standard antimicrobial agents in treating serious infections, and the FDA has warned against the off-label use of tigecycline to treat nosocomial pneumonia because randomized trials have indicated that it confers an increased mortality risk.^39–42^ However, these trials included only a few infections caused by multiple drug-resistant bacteria. Owing to the scarcity of effective drugs for CRE infections, tigecycline should not be incautiously abandoned without comprehensive and objective evaluation.

To our knowledge, this is the first systematic review to assess the efficacy of tigecycline in treating CRE infections. Although the overall mortality did not differ between tigecycline and the control groups, subgroup analysis found the 30-day mortality was significantly lower in the tigecycline combination group than in the control group. As for the clinical and microbiological responses, no significant differences occurred between the 2 groups. The pooled results of the single-arm studies analysis corroborated the findings from the controlled studies. This indicates that tigecycline-based therapy is not *inferior* to other antimicrobial regimens when treating serious CRE infections.

Combination antibiotic therapy for infections caused by carbapenem-resistant Gram-negative bacteria has garnered great interest in recent years. The expanded spectrum of susceptible bacteria, potential for synergistic effects, and reduced incidence of resistance are the main arguments for using combination therapy.^6^ However, no clinical studies have yet shown that synergy improves outcomes, and combination treatment is still controversial.^43^ The present study found that tigecycline combination therapy results in a significantly lower 30-day mortality than does monotherapy or a control. Moreover, the 30-day mortality in the triple tigecycline-containing combinations group was significantly lower than that in the group with dual combinations. These results indicate that tigecycline combination therapy is more effective than monotherapy in treating CRE infections.

In the present study, tigecycline combined with colistin, carbapenems, or aminoglycosides were the most common combination regimens used for CRE infections. However, because our data were limited, we could not assess which combination might be the best choice. In this regard, retrospective cohort studies by Daikos and Tumbarello showed that when carbapenemase-producing *K pneumoniae* had a meropenem MIC of ≤ 8 mg/L, combinations that included meropenem were associated with significantly higher survival rates.^22,32^ Another retrospective study by Gonzalez-Padilla et al found that gentamicin was independently associated with lower 30-day mortality in cases of sepsis caused by CRE.^29^ Hence, more prospective studies are necessary to confirm whether combination regimens including carbapenems and aminoglycosides provide therapeutic benefits.

A major concern regarding the off-label uses of tigecycline to treat serious infections (blood stream infections, urinary tract infections, and nosocomial pneumonia) is the suboptimal drug concentrations that occur at these sites.^8^ Given the pharmacokinetic and pharmacodynamic characteristics of tigecycline, increasing the dose may lead to better clinical outcomes.^10^ In an RCT by Ramirez et al, hospital-acquired pneumonia was cured using 100 mg of tigecycline in 85.0% (17/20) of cases, whereas 75 mg of the same drug cured only 69.6% (16/23) of cases (*P* *=* 0.4).^44^ In the current meta-analysis, pooled data from 2 studies showed that ICU mortality was significantly lower in high-dose groups than in standard-dose groups. Conversely, pooled analysis from 2 further studies showed no difference between the 2 groups in terms of 30-day mortality. However, because of the limited number of patients included in studies, we cannot draw definitive conclusions: the effectiveness of high-dose tigecycline regimens requires further investigation.

Previous studies have shown that the most common adverse effects of tigecycline are gastrointestinal disorders such as nausea, vomiting, and diarrhea.^40^ Three of our single-arm studies monitored adverse effects; in general, tigecycline was well tolerated within the patient populations of the included studies. However, 1 study found that the MICs of tigecycline in 5 different *K pneumoniae* strains increased after tigecycline treatment.^34^ The development of resistance during treatment could lead to treatment failure, as well as the rapid spread of tigecycline-resistant pathogens; this warrants the full attention of all clinicians concerned.

The present systematic review had limitations and should be interpreted with caution. First, none of the included controlled studies were RCTs, and we could not control for some confounding factors (type of patient population, disease severity, site of infection, genotype of pathogens, and cut-off points used for susceptibility testing). Second, the variation in criteria for clinical response may have caused heterogeneity between studies, and the details provided in the studies (time to starting therapy; duration of treatment) were insufficient to allow a more comprehensive interpretation of the results in this regard. Third, in some subgroup analyses, the sample size was small, which may have reduced the power of the statistical analysis.

In conclusion, the present results indicate that tigecycline has a similar efficacy to other antibiotics in treating CRE infections. Combination therapy and high-dose regimens may be superior to monotherapy and standard-dose regimens, respectively. Nonetheless, considering that the current available evidence is limited, well-designed RCTs are urgently needed to clarify the comparative efficacy of tigecycline in treating CRE infections.
